# *Leishmania* spp. in equids and their potential vectors in endemic areas of canine leishmaniasis

**DOI:** 10.1371/journal.pntd.0012290

**Published:** 2024-07-18

**Authors:** Mariaelisa Carbonara, Jairo Alfonso Mendoza-Roldan, Marcos Antônio Bezerra-Santos, Pedro Paulo de Abreu Teles, Riccardo Paolo Lia, Francesco Locantore, Roberta Iatta, Petr Volf, Domenico Otranto

**Affiliations:** 1 Department of Veterinary Medicine, University of Bari, Valenzano, Italy; 2 Interdisciplinary Department of Medicine, University of Bari, Bari, Italy; 3 Department of Parasitology, Faculty of Science, Charles University, Prague, Czech Republic; 4 Department of Veterinary Clinical Sciences, City University of Hong Kong, Hong Kong, SAR China; Insitut Pasteur de Tunis, TUNISIA

## Abstract

Equids may be infected by zoonotic *Leishmania* spp., including *Leishmania infantum*, in regions where canine leishmaniasis (CanL) is endemic, and *Leishmania martiniquensis*, which has been reported in horses from Central Europe. This study was designed to evaluate the occurrence of both *Leishmania* spp. among equids living in CanL endemic areas of Italy, as well as to identify dipteran vectors from the same habitats. From March to October 2023, blood, serum and tissue samples from skin lesions were collected from equids (*n* = 98; *n* = 56 donkeys and *n* = 42 horses) living in Italy, as well as sand flies and biting midges. Blood samples (*n* = 98) and skin lesions (*n* = 56) were tested for *Leishmania* spp. by conventional and real time PCRs and sera were tested by immunofluorescence antibody tests (IFAT) for both *L*. *infantum* and *L*. *martiniquensis*. Insects were morphologically identified, and female specimens (*n* = 268 sand flies, *n* = 7 biting midges) analyzed for *Leishmania* DNA, as well as engorged sand flies (*n* = 16) for blood-meal detection. Two animals with skin lesions (i.e., one donkey and one horse) scored positive for *Leishmania* spp. DNA, and 19 animals (i.e., 19.4%; *n* = 13 donkeys and *n =* 6 horses) were seropositive for *L*. *infantum*, with five of them also for *L*. *martiniquensis*. Most seropositive animals had no dermatological lesions (i.e., 68.4%) while both animals molecularly positive for *Leishmania* spp. scored seronegative. Of the 356 sand flies collected, 12 females (i.e., *n* = 8 *Sergentomyia minuta*; *n* = 3 *Phlebotomus perniciosus*, *n* = 1 *Phlebotomus perfiliewi*) were positive for *Leishmania* spp. DNA, and one out of seven biting midges collected was DNA-positive for *L*. *infantum*. Moreover, engorged sand flies scored positive for human and equine DNA. Data suggest that equids living in CanL endemic areas are exposed to *Leishmania* spp., but their role in the circulation of the parasite needs further investigations.

## Author summary

Horses and donkeys seem to be infected by *Leishmania infantum*, causative agent of canine leishmaniasis (CanL), which is one of the most important zoonotic vector-borne disease. Moreover, *Leishmania martiniquensis*, causing visceral and mucocutaneus leishmaniasis in humans, was reported in horses from central Europe. Therefore, we designed this study to investigate the circulation of both *Leishmania* zoonotic species among equids living in CanL endemic areas and to access the presence of dipteran vectors from the same habitats. Horses and donkeys (*n* = 98) were sampled and analyzed for *Leishmania* spp., as well as sand flies and biting midges. Nineteen equids (19.4%) scored seropositive by immunofluorescence antibody test for *L*. *infantum* and one donkey and one horse, presenting skin lesions, scored molecularly positive for *Leishmania* spp. Twelve sand flies and one biting midge were positive for *Leishmania* spp. DNA. Overall, data herein presented suggest that equids living in CanL endemic areas are exposed to *Leishmania* spp., potentially contributing to the circulation of the parasite.

## Introduction

The leishmaniases include a diverse group of protozoan vector-borne diseases, with around 30 species of *Leishmania* infecting animals and humans [1, 2]. Among them, *Leishmania infantum* is an important species of veterinary and medical relevance, widely distributed in the Mediterranean basin, the Middle East, western Asia and Brazil [3, 4]. This protozoan primarily infects dogs but also a large variety of domestic and wild mammals [4–6], including horses [7]. Indeed, after the first description of *Leishmania* spp. in a horse from Argentina, in the beginning of the 20^th^ century [8], several cases of equine leishmaniasis (EL) have been reported worldwide [9]. Based on results of serological tests, *L*. *infantum*-prevalence ranging from 0.3% to 36% were detected in healthy equids living in countries where canine leishmaniasis (CanL) is endemic, such as Portugal, Italy and Greece [10–13]. In addition, a few cases of cutaneous leishmaniasis due to *L*. *infantum* have been described in horses from Spain, Portugal and Italy [14–16] suggesting that, in areas with prevalent infections in dogs, other animal species may be exposed and/or infected by these parasites [17–19]. Moreover, autochthonous cases of *Leishmania* (*Mundinia*) *martiniquensis*, previously known as ‘*Leishmania siamensis*’, were molecularly detected from skin lesions of horses living in the United States of America [20], Switzerland and Germany [21], where CanL by *L*. *infantum* is not endemic [2]. Importantly, this *Mundinia* species was isolated from human patients in south-east Asia [22,23], being a zoonotic agent of visceral and mucocutaneus leishmaniasis in Thailand [24]. Differently from *L*. *infantum*, many knowledge gaps persist regarding the biology of *L*. *martiniquensis* and their vectors. *Culicoides peregrinus* specimens naturally infected by *L. martiniquensis* were collected in southern Thailand [25], along with laboratory experimental studies demonstrating successful transmission of *Mundinia* species by *Culicoides sonorensis* [26], hence suggesting that biting midges are responsible for *L*. *martiniquensis* transmission in Europe.

Descriptive clinical data about EL are scant, being the infections usually subclinical or characterized by local inflammatory response, with single or multiple papules or ulcerated nodules localized around the eyes, in muzzle, neck, pinnae, scrotum and legs, where biting insects commonly feed [7,9,27]. The diagnosis of EL relies on microscopical observation of amastigotes, usually coupled with the molecular identification of *Leishmania* at the species level [9]. On the other hand, immunofluorescence antibody test (IFAT) and enzyme-linked immunosorbent assay (ELISA) have been employed to detect *Leishmania* exposure in equids [9].

Given all the above, this study aimed to assess the circulation of *L*. *infantum* and *L*. *martiniquensis* among equids living in geographical areas of Italy, endemic for human and CanL, as well as to identify potential dipteran vectors from the same habitats.

## Methods

### Ethics statement

Animals were handled with regard to their well-being and the protocol of this study was approved by the ethical committee of the Department of Veterinary Medicine, University of Bari, Italy (Prot. Uniba 2/24).

### Study areas and equid sample collection

**Fig 1:**
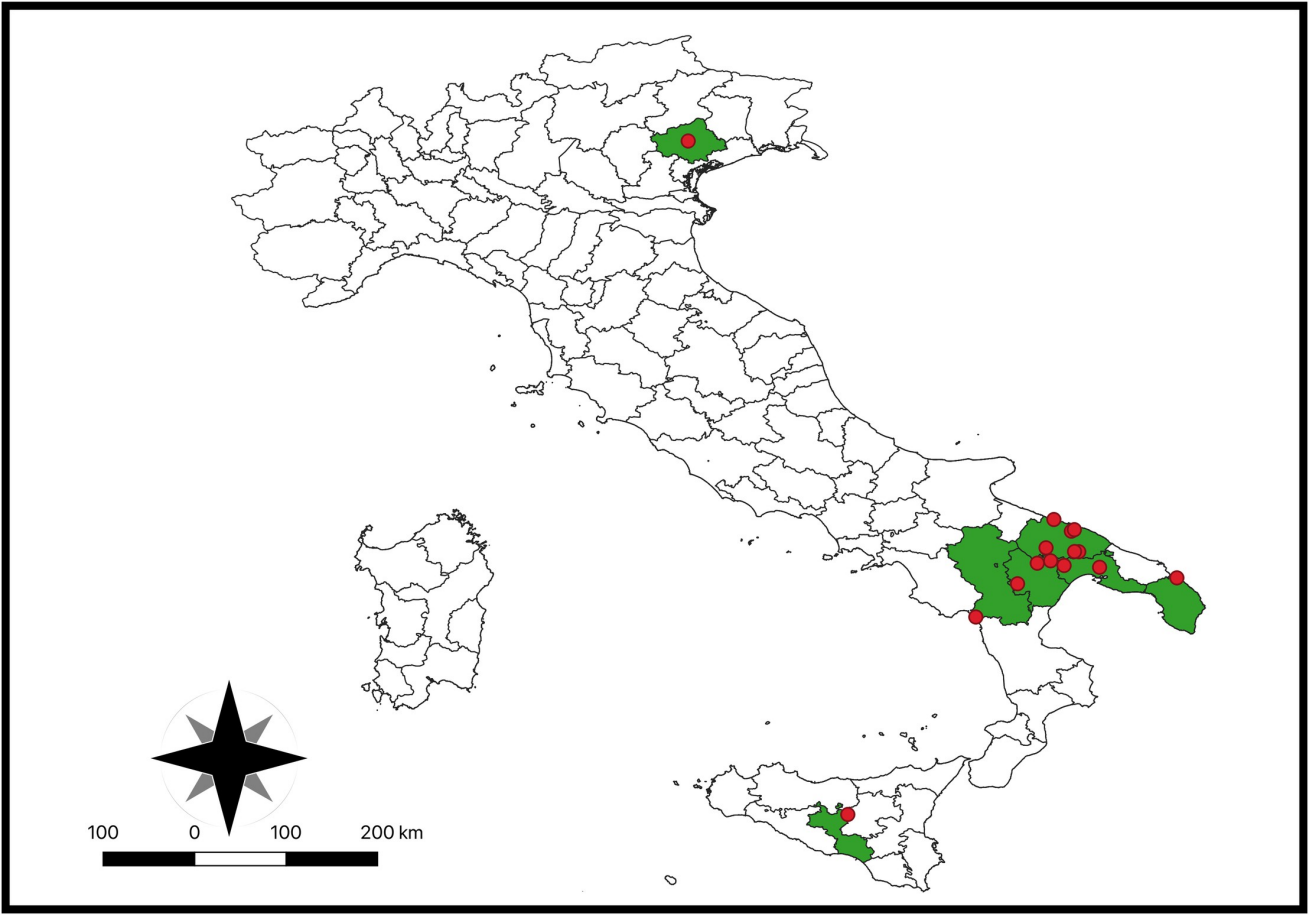
Map of study areas (colored green) indicated by provinces, showing the distribution of equids sampled (red circles). Map prepared using QGIS software—Buenos Aires version (link source of the shapefile: https://www.eea.europa.eu/data-and-maps/data/eea-reference-grids-2/gis-files/italy-shapefile).

**Fig 2:**
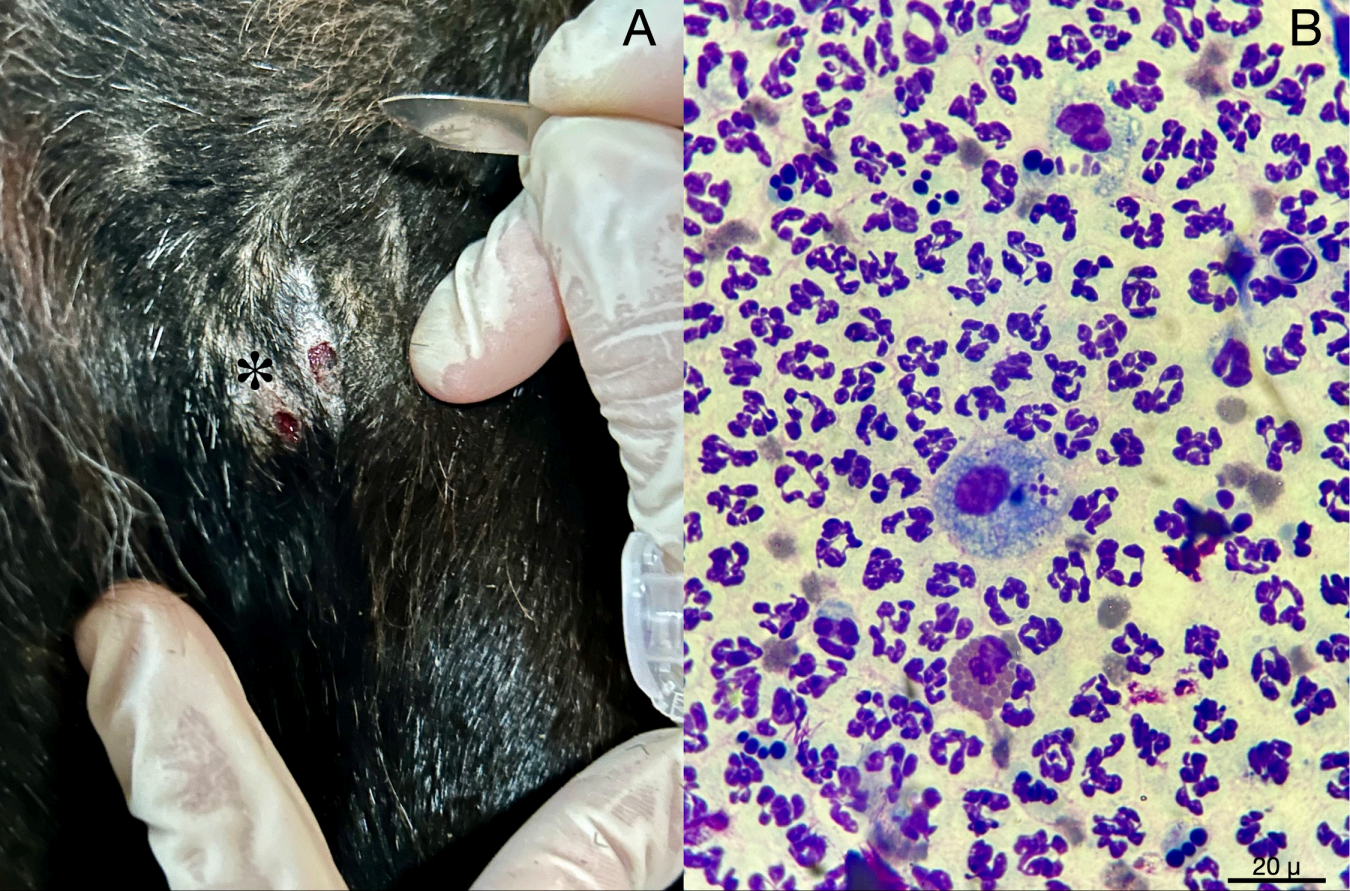
Cytological sampling and processing. A) Donkey presenting two alopecic lesions with epithelial discontinuity. B) Apposition cytology made on the skin lesion (*), with an intense mixed inflammatory infiltrate composed predominantly by neutrophils, activated macrophages phagocytosing neutrophils and apoptotic bodies, and eosinophils. Absence of amastigote forms of *Leishmani*a spp. (Diff Quik stain, 100X).

From March to October 2023, blood and serum were sampled from *n* = 98 equids (i.e., *n* = 56 donkeys and *n* = 42 horses) living in endemic areas for CanL [28], specifically in 10 sites from Apulia, four sites from Basilicata, one from Sicily and one from Veneto regions (Fig 1). Of the 16 sampling sites, 13 were private stables hosting 2–9 animals each, and three were large breeding herds (up to 90 animals each) (Table 1). In addition, when animals presented dermatological signs, biopsies (Fig 2A) and at least three cytological slides were taken from skin lesions (Fig 2B). At the enrollment, animal data (i.e., species, sex, microchip code) along with a brief skin lesion description were recorded in individual files. The location of equid sampling was geo-referenced using a geographical information system (QGIS software, Buenos Aires version).

**Table 1:** Comparison of serological and/or molecular prevalence of *Leishmania* spp. (i.e., *Leishmania infantum* and *Leishmania martiniquensis*) with animal data (i.e., provenience: region, sampling site; animal species: H-horses, D-donkey) and dermatological lesions recorded.

Region	Sampling site	N (Animal species)	N animals with hyperkeratotic and alopecic nodules on the face	N animals with ulcers	N animals with other lesions	qPCR N positive (CT*)	cPCR N positive	IFAT *L*. *infantum* N exposed and positive (titers)	IFAT *L*. *martiniquensis* N exposed and pos (titers)
	**Private stables**								
Veneto	Valdobbiadene	1 H	1	-	1	0	1	0	0
Apulia	Giovinazzo	3 H	1	1	3	0	0	1 (40)	0
Apulia	Triggiano	3 H	3	-	3	0	0	2 (40)	1 (40)
Apulia	Valenzano	2 H	1	-	1	0	0	1 (40)	1 (40)
Apulia	Foresta Mercadante	2 D	1	-	2	0	0	1 (40)	1 (40)
Apulia	Gioia del colle (a)	1 D	1	-	-	0	0	1 (80)	1 (40)
Apulia	Laterza	1 H	-	-	-	0	0	0	0
Basilicata	Contrada Tre Ponti—Matera	9 H	1	-	-	0	0	3 (40); 2 (80)	3 (40)
Basilicata	Stigliano—Matera	1 H	1	-	-	0	0	1 (40)	0
Basilicata	Colle Timmari—Matera	5 H	1	-	-	0	0	1 (40); 2 (80)	1 (40)
Basilicata	Costa Del Fico	3 H	1	-	-	0	0	1 (80)	1 (80)
Apulia	Lecce	1 H	1	-	1	0	0	0	0
Sicily	Stillitano	1 H	1	-	1	0	0	0	0
	**Large breeding herds**								
Apulia	Gioia del colle (b)	12 H	12	-	4	0	0	3 (40); 1 (80)	3 (40)
Apulia	Gioia del colle (c)	10 D	4	-	4	0	0	4 (40); 2 (80)	3 (40); 1 (80)
Apulia	Crispiano, Taranto	43 D	12	1	8	1 (33)	0	8 (40); 9 (80); 1 (160)	16 (40); 3 (80)

*CT: threshold cycle, intersection between the amplification curve and the threshold line

### Serological testing

Serum samples were tested to assess the exposure to *L*. *infantum* and *L*. *martiniquensis*. An IFAT for the detection of IgG anti-*L*. *infantum* was performed adapting the procedures previously described for dogs [29]. Promastigotes of *L*. *infantum* zymodeme MON-1 were used as antigens, while rabbit-anti-horse-IgG FITC (Sigma-Aldrich Chemical, Darmstadt, Germany) diluted at 1:50 was used as conjugate. Each horse serum sample was centrifugated at 1200 rpm for 15 min before starting the IFAT procedure, to reduce the unspecific fluorescence associated with physiological jaundice. To detect the antibodies against *L*. *martiniquensis*, the IFAT was performed using promastigotes of *L*. *martiniquensis* (strain MHOM/TH/2011/CU1) as antigens, following the same procedure for *L*. *infantum* IFAT, except for the use of goat-anti-horse-IgG FITC (Thermo Fisher Scientific, Rockford, USA) diluted 1:1000. For both IFAT tests, serum samples from a horse positive for *L*. *infantum* by molecular analyses (i.e., cPCR), and a healthy horse negative for *L*. *infantum*, were used as positive and negative controls, respectively. Samples were scored as positive when they produced a clear cytoplasmic and membrane fluorescence of promastigotes from a dilution of 1:80, while those that produced promastigote fluorescence at 1:40 were considered as exposed. Positive sera were titrated by serial dilutions until negative results were obtained [30].

### Entomological sampling

**Fig 3:**
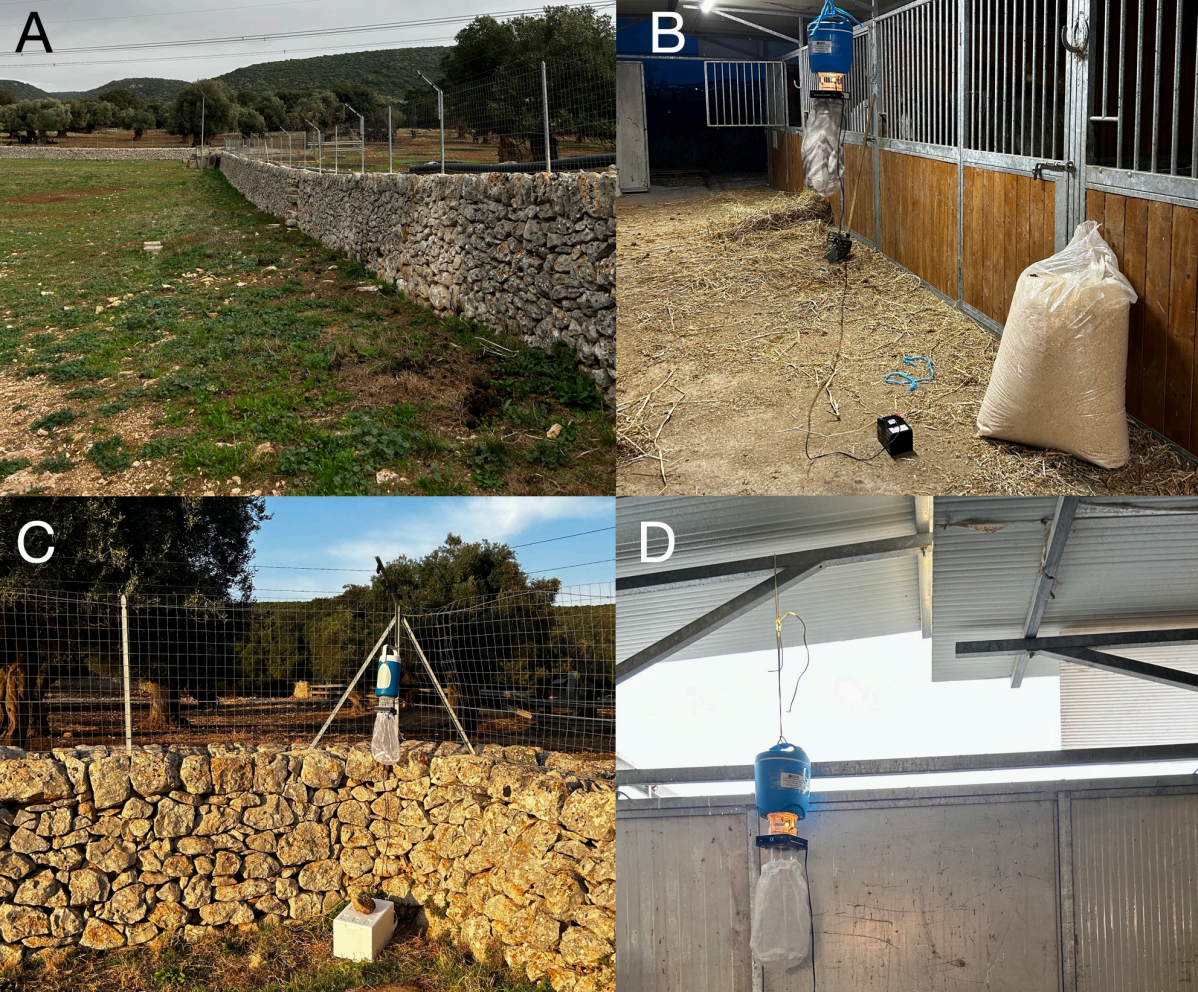
Environmental characteristics of both insect collection sites. A) Large farm with the typical Mediterranean environment (Crispiano, Apulia). B) Small stable, close to the city center (Matera, Basilicata) provided with stalls for equids. C) “Muretto a secco” where the CDC light traps were placed in the Site A. D) Placement of the CDC light traps at the entrance of the stable, Site B.

Sand flies and biting midges were collected from September to October 2023 in two sites (Sites A and B) where *Leishmania*-seropositive equids were found. Site A (Crispiano, Apulia, 40°36′N 17°14′E) was a large farm (i.e., about 80 donkeys and one dog) and had a typical Mediterranean environment characterized by olive trees, the presence of “muretti a secco” (stone walls) where sand flies thrive (Fig 3A); while Site B (Matera, Basilicata, 40°40′N 16°36′E) was a small farm, close to the urban center, with about 7 horses, a donkey and one dog (Fig 3B). Insects were collected on weekly or bi-weekly basis, depending on weather conditions and the availability of the owners, setting from 5:00 p.m. to 8:00 a.m. two CDC light traps with dry ice per site, both outdoor (Fig 3C) and indoor (Fig 3D). Collections were carried out until the total disappearance/absence of insects (i.e., two consecutive negative captures), as previously described [31]. After separating sand flies and biting midges under a stereomicroscope, alive sand fly females were dissected in a drop of saline solution and the gut was observed under an optical microscope to determine the presence of flagellates [32].

### Insect identification and host blood meal detection

Specimens were stored in individual vials containing 70% ethanol and then morphologically identified using taxonomic keys. Heads and last segments of sand flies were removed using sterile tips and mounted on a glass slide using Hoyer’s medium; biting midges were morphologically evaluated based on their wing patterns, head palps and antennae [33–39]. Genomic DNA (gDNA) was extracted from the thorax and abdomen of sand flies (*n* = 268 females and *n* = 5 males) using the GenUP DNA Kit (Biotechrabbit, Berlin, Germany), following the producer’s recommendations. Similarly, the head-thorax of biting midge female specimens (*n* = 7, single sex recorded) were separated from the abdomen using sterile tips and gDNA was extracted using the DNeasy Blood and Tissue Kit (Qiagen GmbH, Hilden, Germany). When sand fly morphological identification was not possible (*n* = 25, *n* = 20 females and *n* = 5 males) due to the absence of morphological relevant segments, molecular identification was carried out by conventional PCR (cPCR), amplifying the mitochondrial DNA fragment encompassing *cytb* and *nd*1 regions (∼500 bp) using the primers PhleF/R, as previously described [40]. Finally, engorged female sand flies (*n* = 16) were tested for blood-meal by cPCR using the primers cytB1-F/B2-R, targeting the vertebrate host mitochondrial *cytb* (350 bp) [41] (Tables 2 and 3). Amplified PCR products were visualized by gel-electrophoresis in 2% agarose gel containing GelRed nucleic acid gel stain (VWR International PBI, Milan, Italy) and viewed on a GelLogic 100 gel documentation system (Kodak, New York, USA). All the positive cPCR products were purified and sequenced in forward direction using the same primers, employing the Big Dye Terminator v.3.1 chemistry in a 3130 Genetic analyzer (Applied Biosystems, Foster city, California, USA) in an automated sequencer (ABI-PRISM 377). Nucleotide sequences were edited, aligned, and analyzed using the Geneious platform version 9.0 (Biomatters Ltd., Auckland, New Zealand) [42], and compared with available sequences in the GenBank database, using the Basic Local Alignment Search Tool (BLAST; http://blast.ncbi.nlm.nih.gov/Blast.cgi) for species identification.

**Table 2:** Species of insects collected according to their site of sampling (i.e., Site A: Crispiano, Apulia; Site B: Matera, Basilicata), sex, feeding status and molecular detection of blood meal. n: number of specimens collected.

Insects	Species (n)	Females	Engorged	Blood meal DNA
**Site A**				
Sand flies	*P*. *perniciosus* (40)	22	3	-
	*P*. *neglectus* (16)	7	1	-
	*S*. *minuta* (86)	75	3	3 *Homo sapiens*
Biting midges	*Culicoides* sp. (1)	1	0	-
	*C*. *pulicaris* complex (1)	1	0	-
	*C*. *catanei* (2)	2	0	-
**Site B**				
Sand flies	*P*. *perniciosus* (120)	112	7	3 *Equus caballus;* 1 *H*. *sapiens*
	*P*. *perfiliewi* (92)	66	2	1 *E*. *caballus*
	*S*. *minuta* (2)	2	0	-
Biting midges	*C*. *obsoletus* complex (1)	1	0	-
	*C*. *imicola* (1)	1	0	-
	*C*. *circumscriptus* (1)	1	0	-

**Table 3:** *Leishmania* spp. (i.e., *Leishmania infantum* or *Leishmania tarentolae*) positivity by quantitative (qPCR/dqPCR) or conventional PCR (cPCR) in insects collected, according to their species, host blood meal detection and site of collection (i.e., Site A: Crispiano, Apulia; Site B: Matera, Basilicata).

Insect	Species	Site	Positive *L*. *infantum* by qPCR (CT*)	Positive *L*. *infantum* by cPCR (primers)	Positive *L*. *tarentolae* by dq PCR (CT*)	Positive *L*. *tarentolae* by cPCR (primers)	Blood meal
Sand fly	*S*. *minuta*	A	yes (32)	yes (L5.8S/LITSR)	no	no	-
	*P*. *perniciosus*	B	yes (34)	no	no	no	*Equus caballus*
	*P*. *perniciosus*	B	yes (37)	no	no	no	-
	*P*. *perniciosus*	B	yes (36)	no	no	no	-
	*P*. *perfiliewi*	B	yes (34)	no	no	no	-
	*S*. *minuta*	A	no	no	yes (24)	yes (F25/R617)	*Homo sapiens*
	*S*. *minuta*	A	no	no	yes (23)	yes (18SN1F/R)	-
	*S*. *minuta*	A	no	no	yes (24)	yes (18SN1F/R)	-
	*S*. *minuta*	A	no	no	yes (22)	yes (18SN1F/R)	-
	*S*. *minuta*	A	no	no	yes (20)	yes (18SN1F/R)	-
	*S*. *minuta*	A	no	no	yes (24)	yes (18SN1F/R)	-
	*S*. *minuta*	B	no	no	yes (24)	yes (18SN1F/R)	-
Biting midge	*C*. *imicola*	B	yes (35)	yes (F25/R617)	no	no	-

*CT: threshold cycle, intersection between the amplification curve and the threshold line

### Molecular detection of *Leishmania*

Genomic DNA was extracted from skin lesions and whole blood (200 μl) using a commercial kit (QIAampDNA Blood Tissue, Qiagen, Hilden, Germany), according to the manufacturer’s instructions. Skin lesion (*n* = 56) and whole blood (*n* = 98) DNA samples, as well as female insect DNA (*n* = 268 sand flies; *n* = 7 biting midges), were tested by real time PCR (qPCR) for the detection of a *L*. *infantum* kDNA minicircle fragment (120 bp), using primers, probes and protocol described elsewhere [43]. In addition, given the sympatric occurrence of *L*. *infantum* and *Leishmania tarentolae* in the areas investigated [31], a duplex real-time PCR (dqPCR) for detection of both *Leishmania* spp. DNA was performed as previously described [44]. Finally, three cPCRs targeting ITS1 (L5.8S/LITSR, ~ 300 bp), 18S rRNA (18SN1F/18SN1R, ~ 290 bp; 18SN2F/18SN2R, ~ 190 bp), and HSP 70 genes (F25/R617, ~ 500 bp) were performed to evaluate the presence of other *Leishmania* species DNA, as previously reported [45–47]. The cPCR protocol by [47] was slightly modified as follows: 95°C for 10 min initial denaturation, followed by 35 cycles of 94°C for 30 sec, 62°C for 30 sec, and 72°C for 30 sec, and then 72°C for 7 min for final elongation. Negative (i.e., male *P*. *perniciosus* specimen) and positive controls (i.e., *L*. *infantum*, *L*. *tarentolae*) were included in all PCR runs. Amplified PCR products were visualized as mentioned above; all the positive cPCR products were purified and sequenced in forward and reverse directions, following the procedures stated overhead.

## Results

### *Leishmania* spp. in equids

**Fig 4:**
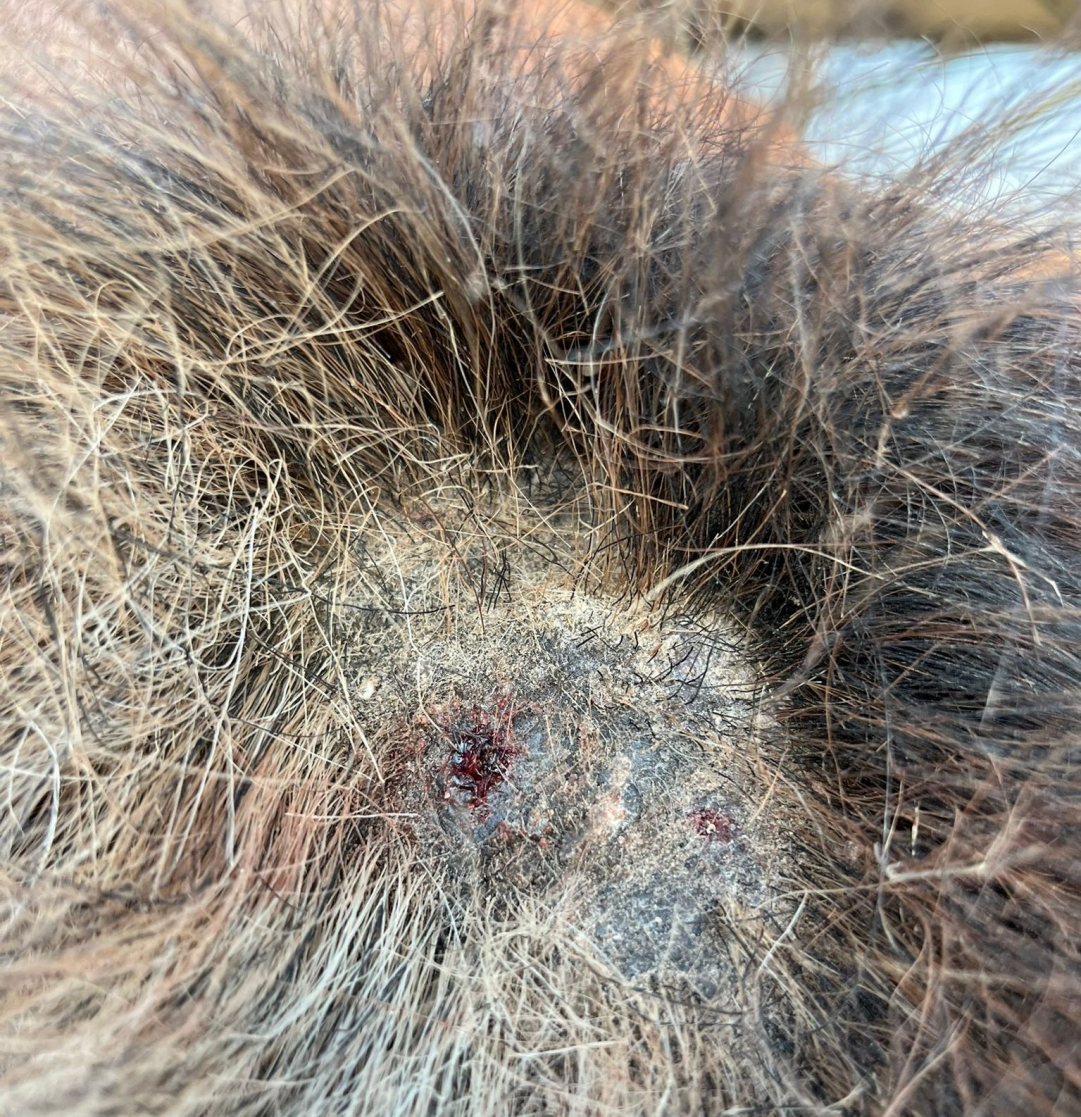
Hyperkeratotic, alopecic, lesion with epithelial discontinuity on the croup of a horse scored molecularly positive to *Leishmania* spp.

Of the sampled equids, 57.1% (i.e., 56/98) presented at least one skin lesion (Fig 4), with hyperkeratotic and alopecic nodules on the face, being the most common dermatological signs (Table 1). While on cytological smear examination, no *Leishmania* spp. amastigotes were observed, one donkey, presenting small nodules on the muzzle, scored positive to *L*. *infantum* by qPCR (i.e., Ct: 33 for both skin lesion tissue and blood), and one horse with hyperkeratotic alopecic area on the tail was positive for *Leishmania* spp. by cPCR (i.e., skin lesion tissue, 18S rRNA, 100% nucleotide identity with GenBank sequences, accession n. MT560279-*Leishmania major*, MK495994-*L*. *infantum*) (Table 1).

Overall, 42.8% (i.e., 42/98) animals had IgG anti-*Leishmania* spp. titers of 1:40 (i.e., *n* = 25 for *L*. *infantum*, *n* = 30 for *L*. *martiniquensis*, of which *n* = 13 for both species). In addition, 19 equids (i.e., *n* = 13 donkeys and *n =* 6 horses; 19.4%) were seropositive for *L*. *infantum* (titers ≥ 1:80) and five of them (i.e., *n =* 1 horse and *n* = 4 donkeys) also for *L*. *martiniquensis* (titers = 1:80) (Table 1). Most seropositive animals had no dermatological lesions (i.e., 68.4%, 12/19) while both animals molecularly positive for *Leishmania* spp. scored seronegative.

### Insect population and *Leishmania* spp. detection

**Fig 5:**
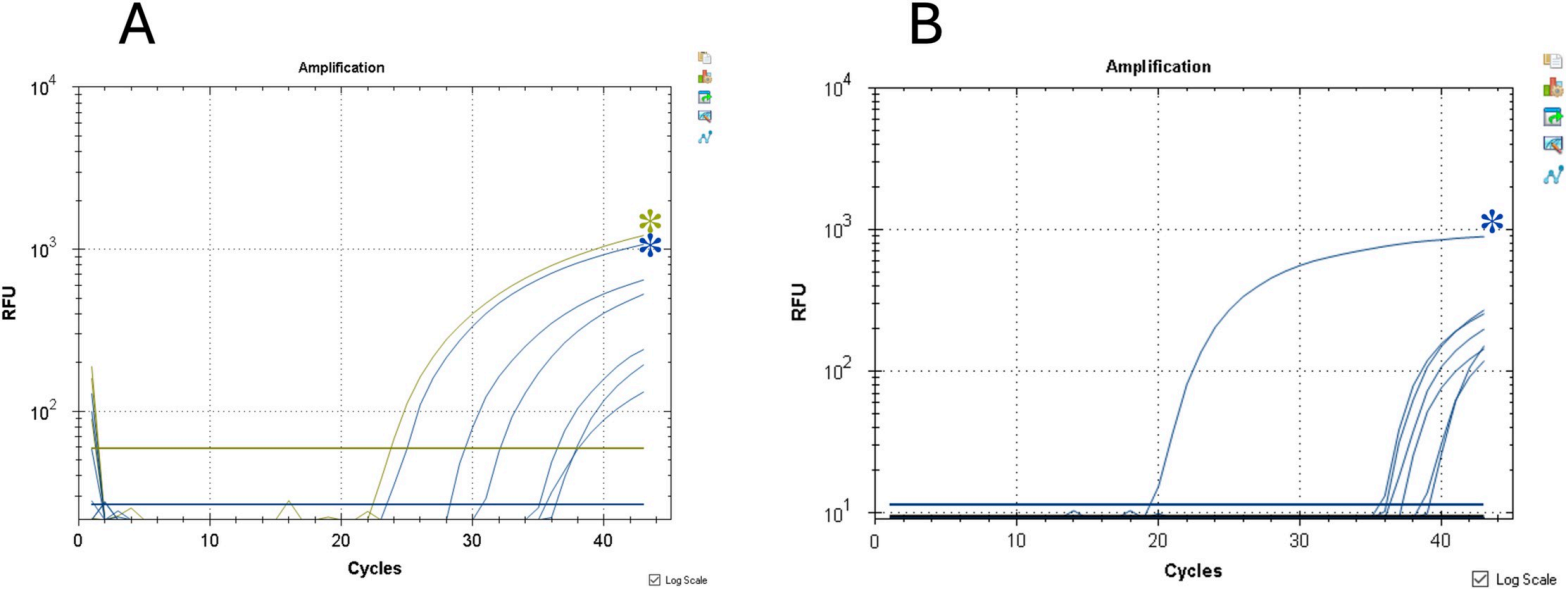
Sand fly qPCR results showing the amplification plot represented by the fluorescent signal, according to relative fluorescence units (RFU) and threshold cycles. A) Duplex qPCR (qdPCR) results with blue colored asterisk and dark green asterisk indicating *Leishmania tarentolae* and *Leishmania infantum* positive controls, respectively. B) qPCR results with blue colored asterisk indicating *L*.*infantum* positive control.

Out of 356 sand flies collected (i.e., *n* = 160 *Phlebotomus perniciosus*, *n* = 92 *Phlebotomus perfiliewi*, *n* = 16 *Phlebotomus neglectus*, *n* = 88 *Sergentomyia minuta*; *n* = 142 from Site A and *n* = 214 from Site B), 284 were females (i.e., *n* = 134 *P*. *perniciosus*, *n* = 66 *P*. *perfiliewi*, *n* = 7 *P*. *neglectus*, and *n* = 77 *S*. *minuta*) (Table 2). While flagellates were not observed during dissection of alive female sand flies, 12 specimens (i.e., 4.5%, 12/268) were molecularly positive for *Leishmania* spp. (i.e., *n* = 7 for *L*. *tarentolae* and *n* = 5 for *L*. *infantum*) (Table 3, Fig 5). Particularly, one *S*. *minuta* from site A scored positive for *L*. *tarentolae* by cPCR (i.e., F25/R617, 100% nucleotide identity with GenBank sequence CP119852) and dqPCR (Ct: 24), being also blood fed on humans (i.e., *Homo sapiens* DNA was detected, 99.7% nucleotide identity with GenBank sequence MG660591). On the other hand, one non-blood fed *S*. *minuta* from the same site was positive for *L*. *infantum* by cPCR (L5.8S/LITSR, 100% nucleotide identity with GenBank sequence MN503527). Similarly, one *P*. *perniciosus* from site B scored positive for *L*. *infantum* by qPCR (Ct: 34), being also blood fed on horse (98.3% nucleotide identity with GenBank sequence MN187576) (Table 3). Of the seven female biting midges collected from sites A and B (i.e., *n* = 1 *Culicoides imicola*, *n* = 1 *Culicoides circumscriptus*, *n* = 2 *Culicoides catanei*, *n* = 1 *Culicoides pulicaris* complex, *n* = 1 *Culicoides obsoletus* complex and *n* = 1 *Culicoides* sp.) (Table 2), one *C*. *imicola* scored positive for *L*. *infantum* by cPCR and qPCR (i.e., F25/R617, 98.83% nucleotide identity with GenBank sequence MW410135 and 99.66% with MH703538; qPCR, Ct: 35) (Table 3).

## Discussion

Horses and donkeys living in endemic areas for CanL are exposed to *Leishmania* spp., being bitten by infected insect vectors. *Leishmania infantum* seroprevalence in equids here recorded with a 1:80 IFAT cut-off value (i.e., 19.4%) was higher than that reported previously in horses from northern/central Italy (i.e., 6.4–13.9%, IFAT, 1:40 dilution cut-off) [12,13], probably because of the higher prevalence of *L*. *infantum* in southern Italian regions [28]. In addition, the high percentage of equids exposed to *Leishmania* spp. (i.e., 42.8%), indicate that the IFAT cut-off of 1:40 is not a reliable positive threshold in *Leishmania* endemic areas, though employed previously [9]. Thus, a 1:80 cut-off value should be recommended as proper IFAT serum dilution to score equids as seropositive to *Leishmania* spp., like previously indicated for cats [48].

Seropositivity for *L*. *infantum* in animals presenting from none to mild-moderate skin disorders, confirms that the infection may cause moderate skin lesions, suggesting that equid immune response is generally effective and prevents the development of systemic disease [49]. Accordingly, *in vitro* cellular immune response demonstrated that horses may develop specific lymphocyte proliferation towards *L*. *infantum*, with limited antibody production, resulting in an effective control of the infection [14,49]. The above might justify the seronegative results in animals scoring molecularly positive to *Leishmania* spp., as previously reported [50]. However, the possibility that equids, at the time of sampling, had already downgraded their *Leishmania*-IgG levels cannot be ruled out, given that the humoral response to *L*. *infantum* in horses can be transient [49].

Since *L*. *martiniquensis* was not molecularly detected in skin lesions of seropositive animals, the IFAT positive results might be due to a cross-reaction between *L*. *infantum* and *L*. *martiniquensis*, as suggested in areas where *L*. *infantum* occurs in sympatry with other species, such as *L*. *tarentolae* [51]. However, the higher exposure recorded for *L*. *martinquensis* than *L*. *infantum* (i.e., 30 animals *vs* 25 animals) may deserve further investigations, also considering that low titers are difficult to be interpreted. Under the above circumstances, the exposure of equids to biting midge saliva, through serological assays, might be important to estimate the risk of *L*. *martiniquensis* infection, as demonstrated for sand fly bites, which are correlated to the risk of *L*. *infantum* infection in dogs and cats [52,53]. Nevertheless, data advocate for molecular tests for EL diagnosis, especially in equids with dermatological lesions, living in or travelled to CanL endemic areas.

The overall *Leishmania* molecular prevalence recorded in sand flies (i.e., 4.5%) falls in ranges of previous records from the same region (i.e., from 4.7% to 12%) [18,31] and it depends on many epidemiological factors at each sampling site as well as on the collecting efforts. The sand fly species composition found is in agreement with data from previous entomological surveys in southern Italy [54,55]. Likewise, the predominance of *S*. *minuta* in Site A and *P*. *perniciosus* in Site B, is consistent with the ecology of these two insect species, the first being more abundant in rural areas and the second in peri-urban settings [56,57]. The finding of human and equine DNA in *S*. *minuta* and *P*. *perniciosus* specimens, respectively, suggest that host choice of these sand flies may depend on hosts’ availability [58]. Accordingly, *P*. *perniciosus* was previously demonstrated to feed on horses in Central Italy [58]. Though the *L*. *infantum-*positive *P*. *perniciosus* found in this study was blood fed on horse, the possibility that the sand fly previously acquired the infection from another host cannot be ruled out. Albeit *S*. *minuta* prefers to feed on reptiles [59,60], its feeding on humans was demonstrated experimentally [59]. The finding of *L*. *infantum* DNA in *S*. *minuta*, the proven vector of *L*. *tarentolae* [60], was previously discussed [18,31] and advocates for future researches aiming to isolate *L*. *infantum* from “unconventional” hosts (i.e., *S*. *minuta* and reptiles), to better understand the epidemiological dynamics driven by the sympatric occurrence of *L*. *infantum* and *L*. *tarentolae*.

The variety of biting midge species identified (i.e., six different species) is consistent with that reported in Italy and other Mediterranean countries (e.g., Morocco, Spain) [38,39,61]. Similarly, *L*. *infantum* DNA was already detected in a wild-caught *C*. *imicola* specimen in Tunisia [62], as well as other species of *Leishmania* from biting midges [63,64]. Noteworthy, the PCR detection of *Leishmania* spp. does not imply the vector capability of any blood sucking insect [65], since only the observation of metacyclic promastigotes in the stomodeal valve or transmission experiments can ensure the vector competence [66].

## Conclusions

Data suggest that equids living in CanL endemic areas are often exposed to *Leishmania* spp., with minor clinical impact. In such areas, horses or donkeys presenting skin lesions should be searched for *Leishmania* spp. molecularly, as this diagnostic method should be preferred to serological ones, due to the cross reactions (e.g., *L*. *infantum vs L*. *martiniquensis*) that may occur at IFAT and ELISA tests. Although *L*. *martiniquensis* was not detected in equids and biting insects in Italy yet, data advocate for a more in-depth evaluation of *Leishmania* species among equids, and in the vector populations from the same geographical areas.

## References

[1] WHO, 2023. Leishmaniasis https://www.who.int/news-room/fact-sheets/detail/leishmaniasis Accessed 14 February 2024

[2] Maia C. Sand fly-borne diseases in Europe: epidemiological overview and potential triggers for their emergence and re-emergence. J Comp Pathol. 2024;209: 6–12. doi: 10.1016/j.jcpa.2024.01.001 38320331

[3] Dantas-Torres F, Solano-Gallego L, Baneth G, Ribeiro VM, de Paiva-Cavalcanti M, Otranto D. Canine leishmaniosis in the Old and New Worlds: unveiled similarities and differences. Trends Parasitol. 2012;28: 531–538. doi: 10.1016/j.pt.2012.08.007 22995719

[4] Baneth G, Solano-Gallego L. Leishmaniasis. Vet Clin North Am Small Anim Pract. 2022;52: 1359–1375. doi: 10.1016/j.cvsm.2022.06.012 36336425

[5] Dantas-Torres. The role of dogs as reservoirs of *Leishmania* parasites, with emphasis on *Leishmania* (Leishmania) *infantum* and *Leishmania* (Viannia) *braziliensis*. Vet. Parasitol. 2007;149: 139–146 17703890 10.1016/j.vetpar.2007.07.007

[6] de Souza NN, Ursine RL, Cruz DS, Xavier EMS, Queiroz LDRP, Falcão LAD, et al. *Leishmania* species infection of bats: A systematic review. Acta Trop. 2023;248: 107025. 37769863 10.1016/j.actatropica.2023.107025

[7] Limeira CH, Alves CJ, Azevedo SS, Santos CSAB, Melo MA, Soares RR, et al. Clinical aspects and diagnosis of leishmaniasis in equids: a systematic review and meta-analysis. Rev Bras Parasitol Vet. 2019;28: 574–581. doi: 10.1590/S1984-29612019074 31596317

[8] Mazza S. Leishmaniasis cutanea en el caballo y nueva observaci n de la misma en el perro. Bol. Univ. B. Aires. 1927;3: 462–464.

[9] Mhadhbi M, Sassi A. Infection of the equine population by *Leishmania* parasites. Equine Vet J. 2020;52: 28–33. 31498914 10.1111/evj.13178

[10] Kouam MK, Diakou A, Kanzoura V, Papadopoulos E, Gajadhar AA, Theodoropoulos G. A seroepidemiological study of exposure to *Toxoplasma*, *Leishmania*, *Echinococcus* and *Trichinella* in equids in Greece and analysis of risk factors. Vet Parasitol. 2010;170: 170–175. 20197215 10.1016/j.vetpar.2010.02.004

[11] Lopes AP, Sousa S, Dubey JP, Ribeiro AJ, Silvestre R, Cotovio M. Prevalence of antibodies to *Leishmania infantum* and *Toxoplasma gondii* in horses from the north of Portugal. Parasites Vectors. 2013;6: 178. 23773870 10.1186/1756-3305-6-178 PMC3686701

[12] Sgorbini M, Bonelli F, Pizzolli I, Tognetti R, Corazza M. Seroprevalence of *Leishmania* sp. infection in healthy horses housed in endemic areas in Tuscany. J. Equine Vet. Sci. 2014;34: 572–574.

[13] Gazzonis AL, Bertero F, Moretta I, Morganti G, Mortarino M, Villa L, et al. Detecting antibodies to *Leishmania infantum* in horses from areas with different epizooticity levels of canine leishmaniosis and a retrospective revision of Italian data. Parasites Vectors. 2020;13: 530. 33092640 10.1186/s13071-020-04385-8 PMC7583181

[14] Solano-Gallego L, Fernandez-Bellon H, Serra R, Gallego M, Ramis A, Fondevila D, et al. Cutaneous leishmaniosis in three horses in Spain. Equine Vet. J. 2003;35: 320–323. doi: 10.2746/042516403776148336 12755438

[15] Gazzonis AL, Morganti G, Porcellato I, Roccabianca P, Avallone G, Gavaudan S, et al. Detection of *Leishmani*a spp. in chronic dermatitis: retrospective study in exposed horse populations. Pathogens. 2022;11: 634. 35745488 10.3390/pathogens11060634 PMC9227255

[16] Gama A, Elias J, Ribeiro AJ, Alegria N, Schallig HD, Silva F, et al. Cutaneous leishmaniosis in a horse from northern Portugal. Vet Parasitol. 2014;200:189–192 doi: 10.1016/j.vetpar.2013.12.005 24388338

[17] Abbate JM, Arfuso F, Napoli E, Gaglio G, Giannetto S, Latrofa MS, et al. *Leishmania infantum* in wild animals in endemic areas of southern Italy. Comp Immunol Microbiol Infect Dis. 2019;67: 101374. 31707163 10.1016/j.cimid.2019.101374

[18] Iatta R, Zatelli A, Laricchiuta P, Legrottaglie M, Modry D, Dantas-Torres F, et al. *Leishmania infantum* in tigers and sand flies from a leishmaniasis-endemic area, Southern Italy. Emerg Infect Dis. 2020;26: 1311–1314. 32441622 10.3201/eid2606.191668 PMC7258470

[19] Cardoso L, Schallig H, Persichetti MF, Pennisi MG. New epidemiological aspects of animal leishmaniosis in europe: the role of vertebrate hosts other than dogs. Pathogens. 2021;10: 307. doi: 10.3390/pathogens10030307 33800782 PMC8000700

[20] Reuss SM, Dunbar MD, Mays MBC, Owen JL, Mallicote MF, Archer LL, et al. Autochthonous *Leishmania siamensis* in horse, Florida, USA. Emerg Infect Dis. 2012;18: 1545–1547. 22932732 10.3201/eid1809.120184 PMC3437729

[21] Müller N, Welle M, Lobsiger L, Stoffel MH, Boghenbor KK, Hilbe M, et al. Occurrence of *Leishmania* sp. in cutaneous lesions of horses in Central Europe. Vet Parasitol. 2009;166: 346–351. 19800739 10.1016/j.vetpar.2009.09.001

[22] Pothirat T, Tantiworawit A, Chaiwarith R, Jariyapan N, Wannasan A, Siriyasatien P, et al. First isolation of *Leishmania* from Northern Thailand: case report, identification as *Leishmania martiniquensis* and phylogenetic position within the *Leishmania enriettii* complex. PLoS Negl Trop Dis. 2014;8: e3339. 25474647 10.1371/journal.pntd.0003339 PMC4256172

[23] Leelayoova S, Siripattanapipong S, Manomat J, Piyaraj P, Tan-Ariya P, Bualert L, et al. Leishmaniasis in Thailand: a review of causative agents and situations. Am J Trop Med Hyg. 2017;96: 534–542. doi: 10.4269/ajtmh.16-0604 28093539 PMC5361524

[24] Srivarasat S, Brownell N, Siriyasatien P, Noppakun N, Asawanonda P, Rattanakorn K, et al. Case Report: autochthonous disseminated cutaneous, mucocutaneous, and visceral leishmaniasis caused by *Leishmania martiniquensis* in a patient with HIV/AIDS from Northern Thailand and literature review. Am J Trop Med Hyg. 2022;107: 1196–1202. 36375453 10.4269/ajtmh.22-0108 PMC9768252

[25] Kaewmee S, Mano C, Phanitchakun T, Ampol R, Yasanga T, Pattanawong U, et al. Natural infection with *Leishmania* (*Mundinia*) *martiniquensis* supports *Culicoides peregrinus* (Diptera: Ceratopogonidae) as a potential vector of leishmaniasis and characterization of a *Crithidia* sp. isolated from the midges. Front Microbiol. 2023;14: 1235254. 37675418 10.3389/fmicb.2023.1235254 PMC10478001

[26] Becvar T, Vojtkova B, Siriyasatien P, Votypka J, Modry D, Jahn P, et al. Experimental transmission of *Leishmania* (*Mundinia*) parasites by biting midges (Diptera: Ceratopogonidae). PLoS Pathog. 2021;17: e1009654. 34115806 10.1371/journal.ppat.1009654 PMC8221790

[27] Matas-Riera M, Cardenas Nadal M, Martínez-Sogues L, Ferrer L. Unilateral keratitis secondary to *Leishmania* spp. infection in a horse: clinical signs and successful topical therapy. Vet Ophthalmol. 2024;27: 86–89. 37489904 10.1111/vop.13134

[28] Mendoza-Roldan J, Benelli G, Panarese R, Iatta R, Furlanello T, Beugnet F, et al. *Leishmania infantum* and *Dirofilaria immitis* infections in Italy, 2009–2019: changing distribution patterns. Parasites Vectors. 2020;13: 193. 32293524 10.1186/s13071-020-04063-9 PMC7161282

[29] Otranto D, Testini G, Dantas-Torres F, Latrofa MS, Diniz PP, de Caprariis D, et al. Diagnosis of canine vector-borne diseases in young dogs: a longitudinal study. J Clin Microbiol. 2010;48: 3316–3324. doi: 10.1128/JCM.00379-10 20660218 PMC2937705

[30] Otranto D, Paradies P, de Caprariis D, Stanneck D, Testini G, Grimm F, et al. Toward diagnosing *Leishmania infantum* infection in asymptomatic dogs in an area where leishmaniasis is endemic. Clin Vaccine Immunol. 2009;16: 337–343. 19129471 10.1128/CVI.00268-08 PMC2650863

[31] Mendoza-Roldan JA, Latrofa MS, Iatta R, R S Manoj R, Panarese R, Annoscia G, et al. Detection of *Leishmania tarentolae* in lizards, sand flies and dogs in southern Italy, where *Leishmania infantum* is endemic: hindrances and opportunities. Parasites Vectors. 2021;14: 461. 34493323 10.1186/s13071-021-04973-2 PMC8423600

[32] Ticha L, Kykalova B, Sadlova J, Gramiccia M, Gradoni L, Volf P. Development of various *Leishmania* (Sauroleishmania) *tarentolae* strains in three *Phlebotomus* species. Microorganisms. 2021;9: 2256. 34835382 10.3390/microorganisms9112256 PMC8622532

[33] Glick JI. *Culicoides* biting midges (Diptera: Ceratopogonidae) of Kenya. J Med Entomol. 1990;27: 85–195. 2093769 10.1093/jmedent/27.2.85

[34] Goffredo M, Meiswinkel R. Entomological surveillance of bluetongue in Italy: methods of capture, catch analysis and identification of *Culicoides* biting midges. Vet Ital. 2004;40: 260–265. 20419674

[35] Talavera S, Muñoz-Muñoz F, Pagès N. New insights on diversity, morphology and distribution of *Culicoides Latreille* 1809 (Diptera: Ceratopogonidae) from Northeast Spain. Ann Soc entomol. Fr. 2011;47: 214–231. Taylor and Francis Group.

[36] Dantas-Torres F, Tarallo VD, Otranto D. Morphological keys for the identification of Italian phlebotomine sand flies (Diptera: Psychodidae: Phlebotominae). Parasites Vectors. 2014;7: 479. doi: 10.1186/s13071-014-0479-5 25323537 PMC4203899

[37] Nielsen SA, Kristensen M. Delineation of *Culicoides* species by morphology and barcode exemplified by three new species of the subgenus *Culicoides* (Diptera: Ceratopogonidae) from Scandinavia. Parasites Vectors. 2015;8: 151. 25889579 10.1186/s13071-015-0750-4 PMC4372322

[38] Augot D, Mathieu B, Hadj-Henni L, Barriel V, Zapata Mena S, Smolis S, et al. Molecular phylogeny of 42 species of *Culicoides* (Diptera, Ceratopogonidae) from three continents. Parasite. 2017;24: 23. 28643630 10.1051/parasite/2017020 PMC5482051

[39] Bourquia M, Garros C, Rakotoarivony I, Gardès L, Huber K, Boukhari I, et al. Update of the species checklist of *Culicoides Latreille*, 1809 biting midges (Diptera: Ceratopogonidae) of Morocco. Parasites Vectors. 2019;12: 459. 31551074 10.1186/s13071-019-3720-4 PMC6757417

[40] Latrofa MS, Dantas-Torres F, Weigl S, Tarallo VD, Parisi A, Traversa D, et al. Multilocus molecular and phylogenetic analysis of phlebotomine sand flies (Diptera: Psychodidae) from southern Italy. Acta Trop. 2011;119: 91–98. doi: 10.1016/j.actatropica.2011.04.013 21635869

[41] Maia C, Parreira R, Cristóvão JM, Freitas FB, Afonso MO, Campino L. Molecular detection of *Leishmania* DNA and identification of blood meals in wild caught phlebotomine sand flies (Diptera: Psychodidae) from southern Portugal. Parasites Vectors. 2015;8: 173. 25889732 10.1186/s13071-015-0787-4 PMC4377202

[42] Kearse M, Moir R, Wilson A, Stones-Havas S, Cheung M, Sturrock S, et al. Geneious Basic: an integrated and extendable desktop software platform for the organization and analysis of sequence data. Bioinformatics. 2012;28: 1647–1649. doi: 10.1093/bioinformatics/bts199 22543367 PMC3371832

[43] Francino O, Altet L, Sánchez-Robert E, Rodriguez A, Solano-Gallego L, Alberola J, et al. Advantages of real-time PCR assay for diagnosis and monitoring of canine leishmaniosis. Vet Parasitol. 2006;137: 214–221. doi: 10.1016/j.vetpar.2006.01.011 16473467

[44] Latrofa MS, Mendoza-Roldan JA, Manoj RRS, Pombi M, Dantas-Torres F, Otranto D. A duplex real-time PCR assay for the detection and differentiation of *Leishmania infantum* and *Leishmania tarentolae* in vectors and potential reservoir hosts. Entomol Gen. 2021;41: 543–551.

[45] El Tai NO, El Fari M, Mauricio I, Miles MA, Oskam L, El Safi SH, et al. *Leishmania donovani*: intraspecific polymorphisms of Sudanese isolates revealed by PCR-based analyses and DNA sequencing. Exp Parasitol. 2001;97: 35–44. 11207112 10.1006/expr.2001.4592

[46] Van der Auwera G, Maes I, De Doncker S, Ravel C, Cnops L, Van Esbroeck M, et al. Heat-shock protein 70 gene sequencing for *Leishmania* species typing in European tropical infectious disease clinics. Euro Surveill. 2013;18: 20543. 23929181 10.2807/1560-7917.es2013.18.30.20543

[47] Sadlova J, Bacikova D, Becvar T, Vojtkova B, England M, Shaw J, Volf P. *Porcisia* transmission by prediuresis of sand flies. Front Cell Infect Microbiol. 2022;12: 981071. 36034718 10.3389/fcimb.2022.981071 PMC9399930

[48] Pennisi MG, Cardoso L, Baneth G, Bourdeau P, Koutinas A, Miró G, et al. LeishVet update and recommendations on feline leishmaniosis. Parasites Vectors. 2015;8: 302. doi: 10.1186/s13071-015-0909-z 26041555 PMC4462189

[49] Fernàndez-Bellon H, Solano-Gallego L, Bardagí M, Alberola J, Ramis A, Ferrer L. Immune response to *Leishmania infantum* in healthy horses in Spain. Vet Parasitol. 2006;135: 181–185. 16213661 10.1016/j.vetpar.2005.09.007

[50] Koehler K, Stechele M, Hetzel U, Domingo M, Schönian G, Zahner H, et al. Cutaneous leishmaniosis in a horse in southern Germany caused by *Leishmania infantum*. Vet Parasitol. 2002;109: 9–17. 12383621 10.1016/s0304-4017(02)00246-7

[51] Iatta R, Carbonara M, Morea A, Trerotoli P, Benelli G, Nachum-Biala Y, et al. Assessment of the diagnostic performance of serological tests in areas where *Leishmania infantum* and *Leishmania tarentolae* occur in sympatry. Parasites Vectors. 2023;16: 352. 37807047 10.1186/s13071-023-05981-0 PMC10561492

[52] Kostalova T, Lestinova T, Sumova P, Vlkova M, Rohousova I, Berriatua E, et al. Canine antibodies against salivary recombinant proteins of *Phlebotomus perniciosus*: a longitudinal study in an endemic focus of canine Leishmaniasis. PLoS Negl Trop Dis. 2015;9: e0003855. 26111018 10.1371/journal.pntd.0003855 PMC4482481

[53] Pereira A, Cristóvão JM, Vilhena H, Martins Â, Cachola P, Henriques J, et al. Antibody response to *Phlebotomus perniciosus* saliva in cats naturally exposed to phlebotomine sand flies is positively associated with *Leishmania* infection. Parasites Vectors. 2019;12: 128. 30909940 10.1186/s13071-019-3376-0 PMC6434892

[54] Tarallo VD, Dantas-Torres F, Lia RP, Otranto D. Phlebotomine sand fly population dynamics in a leishmaniasis endemic peri-urban area in southern Italy. Acta Trop. 2010;116: 227–234. doi: 10.1016/j.actatropica.2010.08.013 20816927

[55] Dantas-Torres F, Tarallo VD, Latrofa MS, Falchi A, Lia RP, Otranto D. Ecology of phlebotomine sand flies and *Leishmania infantum* infection in a rural area of southern Italy. Acta Trop. 2014;137: 67–73. 24813871 10.1016/j.actatropica.2014.04.034

[56] Rossi E, Bongiorno G, Ciolli E, Di Muccio T, Scalone A, Gramiccia M, et al. Seasonal phenology, host-blood feeding preferences and natural *Leishmania* infection of *Phlebotomus perniciosus* (Diptera, Psychodidae) in a high-endemic focus of canine leishmaniasis in Rome province. Italy Acta Trop. 2008;105: 158–165. 18035329 10.1016/j.actatropica.2007.10.005

[57] Maroli M, Feliciangeli MD, Bichaud L, Charrel RN, Gradoni L. Phlebotomine sandflies and the spreading of leishmaniases and other diseases of public health concern. Med Vet Entomol. 2013;27: 123–147. doi: 10.1111/j.1365-2915.2012.01034.x 22924419

[58] Bongiorno G, Habluetzel A, Khoury C, Maroli M. Host preferences of phlebotomine sand flies at a hypoendemic focus of canine leishmaniasis in central Italy. Acta Trop. 2003; 88:109–16. doi: 10.1016/s0001-706x(03)00190-6 14516922

[59] Ticha L, Volfova V, Mendoza-Roldan JA, Bezerra-Santos MA, Maia C, Sadlova J, et al. Experimental feeding of *Sergentomyia minuta* on reptiles and mammals: comparison with *Phlebotomus papatasi*. Parasite Vectors. 2023;16: 126. 10.1186/s13071-023-05758-5 PMC10103492 37055860

[60] Klatt S, Simpson L, Maslov DA, Konthur Z. *Leishmania tarentolae*: Taxonomic classification and its application as a promising biotechnological expression host. PLoS Negl Trop Dis. 2019;13: e0007424. 31344033 10.1371/journal.pntd.0007424 PMC6657821

[61] Quaglia M, Foxi C, Satta G, Puggioni G, Bechere R, De Ascentis M, et al. *Culicoides* species responsible for the transmission of Epizootic Haemorrhagic Disease virus (EHDV) serotype 8 in Italy. Vet Ital. 2023;59: 83–89. 10.12834/VetIt.3347.22208.1 37731311

[62] Slama D, Haouas N, Remadi L, Mezhoud H, Babba H, Chaker E. First detection of *Leishmania infantum* (Kinetoplastida: Trypanosomatidae) in *Culicoides* spp. (Diptera: Ceratopogonidae). Parasites Vectors. 2014;7: 51. 24460752 10.1186/1756-3305-7-51 PMC3906888

[63] Rebêlo JM, Rodrigues BL, Bandeira MD, Moraes JL, Fonteles RS, Pereira SR. Detection of *Leishmania amazonensis* and *Leishmania braziliensis* in *Culicoides* (Dipter Ceratopogonidae) in an endemic area of cutaneous leishmaniasis in the Brazilian Amazonia. J Vector Ecol. 2016;41: 303–308. 27860021 10.1111/jvec.12227

[64] Ríos-Tostado JJ, Castillo-Ureta H, Torres-Montoya EH, Torres-Avendaño JI, Olimo´n-Andalo´n V, Romero-Higareda, et al. Molecular detection of *Leishmania* (*L*.) *mexicana* (Kinetoplastida: Trypanostomatidae) DNA in *Culicoides furens* (Diptera: Ceratopogonidae) from an area with autochthonous canine leishmaniasis in Northwestern Mexico. Acta Parasitol. 2021;66: 1055–1058. 33554301 10.1007/s11686-021-00335-1

[65] Seblova V, Sadlova J, Carpenter S, Volf P. Speculations on biting midges and other bloodsucking arthropods as alternative vectors of *Leishmania*. Parasites Vectors. 2014;7: 222. 24884857 10.1186/1756-3305-7-222 PMC4024269

[66] Dostálová A, Volf P. *Leishmania* development in sand flies: parasite-vector interactions overview. Parasites Vectors. 2012;5: 276. 23206339 10.1186/1756-3305-5-276 PMC3533922

